# Synthesis and Antifungal Activity of Novel Sulfone Derivatives Containing 1,3,4-Oxadiazole Moieties

**DOI:** 10.3390/molecules16119129

**Published:** 2011-11-01

**Authors:** Weiming Xu, Jiang He, Ming He, Feifei Han, Xuehai Chen, Zhaoxi Pan, Jian Wang, Maoguo Tong

**Affiliations:** State Key Laboratory Breeding Base of Green Pesticide and Agricultural Bioengineering, Key Laboratory of Green Pesticide and Agricultural Bioengineering, Ministry of Education, Center for Research and Development of Fine Chemicals, Guizhou University, Guiyang 550025, China; Email: he6701324jiang@163.com (J.H.); hmher@126.com (M.H.); yougubaihe101@163.com (F.H.); chenxuehai2010@foxmail.com (X.C.); panpan0301-121@163.com (Z.P.); wangjmy2008@126.com (J.W.); tongmaoguo@163.com (M.T.)

**Keywords:** sulfone, oxadiazole, antifungal activity

## Abstract

A series of new sulfone compounds containing 1,3,4-oxadiazole moieties were synthesized. The structures of these compounds were confirmed by spectroscopic data (IR, ^1^H- and ^13^C-NMR) and elemental analyses. Antifungal tests indicated that all the title compounds exhibited good antifungal activities against eight kinds of plant pathogenic fungi, and some showed superiority over the commercial fungicide hymexazol. Among them, compounds **5d**, **5e**, **5f**, and **5i** showed prominent activity against *B. cinerea*, with determined EC_50_ values of 5.21 μg/mL, 8.25 µg/mL, 8.03 µg/mL, and 21.00 µg/mL, respectively. The present work demonstrates that sulfone derivatives such as **5d**containing a 1,3,4-oxadiazole moiety can be used as possible lead compounds for the development of potential agrochemicals.

## 1. Introduction

The emergence of fungal resistance to existing fungicides has posed a serious concern for pesticide professionals during the last decade, and the desire for safer and more effective agrochemicals with reduced environmental toxicity also remains a high priority [1]. A further aim is to produce novel fungicides that do not impede the role of beneficial organisms in plant development and which do not persist in the environment and food chains [2], so the synthesis and antifungal evaluation of new compounds is greatly needed.

In this context, sulfone derivatives provide an example of an important class of bioactive compounds with a wide spectrum of activities, as the sulfone group is an important core found in numerous biologically active compounds with a wide range of biological activity including insecticidal [3], antifungal [4], herbicidal [5], anti-hepatitis [6], antitumor [7], anti-inflammatory [8], anticancer [9], anti-HIV-1 [10] and anti-tubercular [11] properties. There is evidence that the key feature of these compounds is a 5- or 6-membered heterocyclic ring attached to a sulfone, and additional modification of the heterocyclic ring has been considered. Among these derivatives, a 2-((4-chlorobenzyl)sulfonyl)-5-(methylsulfonyl)-1,3,4-thiadiazole sulfone prepared by Joachim *et al*. exhibited good inhibitory activity against *Plasmopara viticola * [12] at a concentration of 1 × 10^−5^ mg/kg, 2,4-dibromo-5-methyl-1-((2-methyl-5-nitrophenyl)sulfonyl)-1*H*-imidazole sulfone, reported by Assmann *et al.*, exhibited strong activity against *Phytophthora infestans* and *Plasmopara viticola* [13] at a concentration of 50 g/ha, and 2-(5-ethyl-1-methyl-1*H*-pyrazol-3-yl)-5-(methylsulfonyl)-1,3,4-oxadiazole sulfone, prepared by Yuan *et al.*, exhibited medium inhibitory activity against *Phoma asparagi* [14]. As an illustration of a practical application, the agricultural fungicide oxycarboxin was successfully commercialized by Uniroyal Co. in the year 1966. In the past few decades, a large number of other fungicides with potent bioactivity containing sulfone units such as tolylfluanid, dichlofluanid, cyazofamid, amisulbrom and oryzaemate have been introduced in the market by various companies [15,16].

The 1,3,4-oxadiazole scaffold is an important pharmacophore in agricultural science and compounds bearing this moiety often display antifungal [17], herbicidal [18] and insecticidal [19,20] activities. As a illustration of the activity of 1,3,4-oxadiazole sulfones, Keshari *et al*. reported that 2-(5-sulfanyl-1,3,4-oxadiazol-2-yl)phenylacetate and 5-(pyridin-3-yl)-1,3,4-oxadiazole-2-thiol exhibit good antibacterial activities against *Escherichia coli *(MTCC 443) [21].

As part of our ongoing search for novel sulfone compounds possessing antifungal properties, new derivatives of 2-sulfonyl-5-(3,4,5-trimethoxyphenyl)-1,3,4-oxadiazole (**I**) [22] and 2-sulfinyl-5-(3,4,5-trimethoxyphenyl)-1,3,4-oxadiazole (**II**) [23] were synthesized. Subsequent *in vitro* bioassays disclosed that the compounds 2-(methylsulfonyl)-5-(3,4,5-trimethoxyphenyl)-1,3,4-oxadiazole (**Ia**) and 2-(benzylsulfinyl)-5-(3,4,5-trimethoxyphenyl)-1,3,4-oxadiazole (**IIa**) possess high antifungal activities against 10 kinds of fungi, with EC_50_ values ranging from 19.9 μg/mL to 93.3 μg/mL, being equivalent or more potent against the tested fungi than the commercial agricultural fungicide hymexazol, and they also showed broad-spectrum bioactivity.

**Scheme 1:**
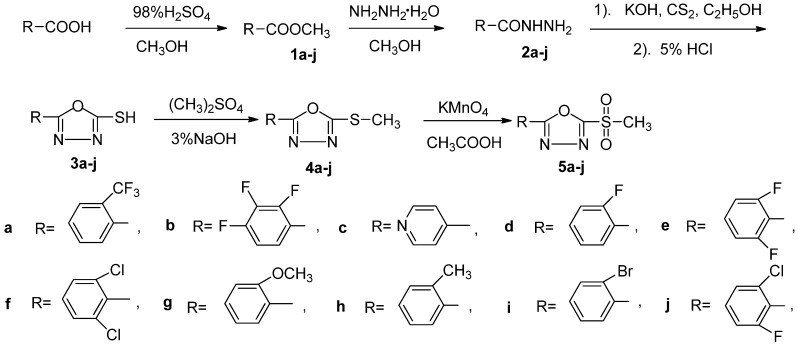
Synthetic route to the title compounds.

The SAR suggested that 2-(methylsulfonyl)-1,3,4-oxadiazole is the core ring system that affords potent antifungal activities [22,23]. Given the apparent lack of scope for changes to the core ring system, the majority of our efforts were directed towards compounds of the general structure of the title compounds in which the core ring system was kept constant and the peripheral groups were altered. As a consequence, in an attempt to increase the fitting to the pharmacophoric model, and possibly to obtain new fungicides, we report here the synthesis, characterization and antifungal activity of novel sulfone derivatives containing 1,3,4-oxadiazole moieties 5, as shown in Scheme 1.

## 2. Results and Discussion

### 2.1. Chemistry Benzohydrazide

The key intermediate 2-thiol-5-substituted-1,3,4-oxadiazole (**3**) was prepared by cyclization of substituted phenylhydrazide, potassium hydroxide, and carbon disulfide in ethanol under reflux conditions. The key point of this reaction is that water must be removed completely; as the presence of even a little water may cause the cyclization to fail.

Although the electron rich methylthio moiety in compounds **4** can be oxidized to a sulfone by a variety of agents such as *m*-CPBA [24] or H_2_O_2_catalyzed by methyltrioxorhenium [25], unfortunately, most of these reagents are not satisfactory. They are either harmful or expensive, and a simple procedure is not easily available. In this experiment, the methylthio moiety in **4** was oxidized with potassium permanganate [26] in glacial acetic acid to afford the corresponding methylsulfonyl species **5**, the advantage of this oxidation is that it uses an inexpensive oxidant without a catalyst; furthermore, the reaction is quickly complete and easy to work up. The physical characteristics, IR, ^1^H-NMR, ^13^C-NMR and elemental analyses data for all the synthesized compounds are reported in the Experimental section.

### 2.2. The Antifungal Activities of Oxadiazole Methyl Sulfones

The inhibitory effects of the synthesized oxadiazole methyl sulfone compounds on phytopathogenic fungi was studied. Two fungi, *F. oxysporum* and *C. mandshurica*, representing typical fungi often occurring in the Chinese agro-ecosystem were chosen for fungicide screening using the mycelial growth rate method. The results were compared with that of the commercial agricultural fungicide hymexazol (a broad spectrum fungicide), as indicated in Table 1.

**Table 1:** Inhibition effect of oxadiazole methyl sulfones against phytopathogenic fungi at 50 µg/mL. 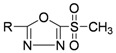

Compound	R	Inhibition (%)	
		*F. oxysporum*	*C. mandshurica*
**5a**	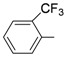	71.1 ± 7.7	68.3 ± 9.3
**5b**	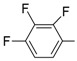	97.5 ± 3.3	89.7 ± 3.1
**5c**	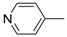	72.6 ± 6.4	78.2 ± 4.7
**5d**	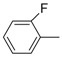	70.1 ± 4.9	64.0 ± 1.3
**5e**	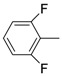	98.8 ± 8.0	97.8 ± 11.8
**5f**	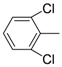	94.0 ± 4.1	97.6 ± 4.3
**5g**	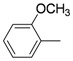	89.6 ± 4.5	91.3 ± 9.6
**5h**	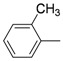	74.6 ± 8.5	99.3 ± 12.8
**5i**	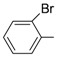	77.0 ± 6.6	79.6 ± 7.1
**5j**	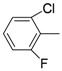	67.1 ± 4.8	64.0 ± 2.6
**Hymexazol**		58.4 ± 0.8	57.3 ± 0.3

As indicated in Table 1, at the concentration of 50 µg/mL, all of the tested compounds exhibited good inhibitory effects against *F. oxysporum*, and all of them showed superiority over the commercial fungicide hymexazol. Among them, compounds **5b**, **5e** and **5f** almost completely inhibited the growth of *F. oxysporum*, with control efficacies of 97.5%, 98.8%, and 94.0%, respectively. Compound **5g** showed 89.6% fungicidal activity against *F. oxysporum*, while the others had inhibition activities between 67.1% and 77.0%. As the results described in Table 1 indicate, all of the tested compounds possessed promising inhibitory effects against *C. mandshurica*. Among them, compounds **5e**, **5f**, **5g**, and **5h** almost completely inhibited the growth of *C. mandshurica*, with inhibition values of more than 90.0%, whereas compound **5b** showed 89.7% fungicidal activity against *C. mandshurica*, and compounds **5a**, **5c**, **5d**, **5i**, and **5j** inhibited the growth of *C. mandshurica* by 68.3%, 78.2%, 64.0%, 79.6%, and 64.0%, respectively.

### 2.3. Toxicity of Some Title Compounds on 8 Kinds of Pathogenic Fungi

We choose some representative compounds with good, ordinary antifungal activity, as indicated in the previous bioassays, to conduct further work which disclosed that some sulfone compounds containing a methyl oxadiazole showed remarkable inhibitory effect on eight kinds of plant pathogenic fungi, which represent typical fungi often occurring in the Chinese agro-ecosystem. The results are summarized in Table 2.

**Table 2:** Toxicity of some methyl sulfones on eight kinds of pathogenic fungi.

Compounds	Fungi	Toxic regression equation	EC_50_ (µg/mL)	R
**5d **	*C. mandshurica*	y = 1.428x + 2.283	79.92 ± 14.79	0.861
	*F. oxysporum*	y = 1.355x + 3.017	29.07 ± 7.82	0.952
	*R. solani *	y = 2.163x + 3.251	6.43 ± 1.34	0.878
	*B. cinerea*	y = 1.341x + 4.038	5.21 ± 2.05	0.921
	*P. infestans*	y = 1.372x + 3.397	14.73 ± 3.23	0.846
	*C. gloeosporioides*	y = 2.930x + 1.173	20.23 ± 6.65	0.961
	*S. sclerotiorum*	y = 1.860x + 3.272	8.49 ± 3.51	0.919
	*T. cucumeris*	y = 3.537x + 1.460	10.01 ± 5.64	0.974
**5e **	*C. mandshurica*	y = 3.623x − 0.735	38.27 ± 3.21	0.867
	*F. oxysporum*	y = 1.439x + 2.384	65.75 ± 7.04	0.976
	*T. cucumeris*	y = 7.95x − 5.878	23.35 ± 4.76	0.980
	*R. solani *	y = 3.681x + 0.115	21.23 ± 4.12	0.916
	*B. cinerea*	y = 1.993x + 3.173	8.25 ± 0.85	0.853
	*P. infestans*	y = 1.216x + 2.842	59.52 ± 16.79	0.991
	*C. gloeosporioides*	y = 4.629x − 1.556	26.07 ± 7.32	0.943
	*S. sclerotiorum*	y = 5.984x − 2.034	14.97 ± 6.83	0.974
**5f **	*C. mandshurica*	y = 1.131x + 2.747	98.18 ± 8.35	0.981
	*F. oxysporum*	y = 1.081x + 2.912	85.41 ± 17.92	0.988
	*T. cucumeris*	y = 2.381x + 1.661	25.25 ± 2.34	0.911
	*R. solani *	y = 2.432x + 2.061	16.16 ± 9.76	0.916
	*B. cinerea*	y = 2.528x + 2.712	8.03 ± 0.86	0.962
	*P. infestans*	y = 1.163x + 3.101	42.93 ± 7.38	0.993
	*C. gloeosporioides*	y = 1.861x + 2.171	33.12 ± 8.29	0.979
	*S. sclerotiorum*	y = 5.036x − 1.223	17.20 ± 4.72	0.951
**5i **	*F. oxysporum*	y = 4.243x − 1.261	29.89 ± 1.31	0.918
	*C. mandshurica*	y = 4.355x − 2.179	44.50 ± 3.56	0.947
	*R. solani *	y = 5.036x − 1.879	20.02 ± 1.28	0.978
	*T. cucumeris*	y = 5.285x − 3.994	24.78 ± 4.29	0.964
	*S. sclerotiorum*	y = 2.562x + 2.003	14.78 ± 1.02	0.879
	*B. cinerea*	y = 7.582x − 5.026	21.00 ± 2.01	0.947
	*C. gloeosporioides*	y = 6.364x − 3.537	21.95 ± 2.93	0.963
	*P. infestans*	y = 1.358x + 2.697	49.64 ± 9.39	0.958
Hymexazol	*F. oxysporum*	y = 1.343x + 3.058	27.93 ± 1.02	0.980
	*C. mandshurica*	y = 2.103x + 1.647	39.26 ± 2.79	0.999
	*R. solani *	y = 3.532x − 0.604	38.64 ± 0.45	0.880
	*T. cucumeris*	y = 1.298x + 3.043	32.21 ± 5.82	0.958
	*S. sclerotiorum*	y = 2.346x + 2.900	7.76 ± 2.98	0.998
	*C. gloeosporioides*	y = 3.896x − 1.136	37.58 ± 3.16	0.946
	*P. infestans*	y = 1.715x + 2.559	26.49 ± 1.42	0.858
	*B. cinerea*	y = 2.014x + 2.177	25.23 ± 6.12	0.917

As indicated in Table 2, all of the test compounds possessed prominent antifungal activities against eight plant pathogens (*F. oxysporum*, *C. mandshurica*, *R. solani*, *T. cucumeris*, *S. sclerotiorum*, *C. gloeosporioides*, *P. infestans*, *B. cinerea*), with EC_50_ values between 5.21 µg/mL to 98.18 µg/mL. Among them, the bioassay results showed that compounds **5d**, **5e**, **5f**, and **5i** showed prominent activity against *R. solani* (EC_50_ values of 6.43 µg/mL, 21.23 µg/mL, 16.16 µg/mL, and 20.02 µg/mL, respectively), which was superior to the activity of the commercial fungicide hymexazol (38.64 µg/mL). *B. cinerea *has been identified as a pathogen of more than 235 plant species, including grapes, lettuce, tomatoes, tobacco, and strawberries, producing a gray powdery mold on the infected crops. To our delight, compounds **5d**, **5e**, **5f**, and **5i** showed prominent activity against *B. cinerea*, the EC_50_values of 5.21 µg/mL, 8.25 µg/mL, 8.03 µg/mL, and 21.00 µg/mL, respectively, all of them being more effective than the positive control. The results also suggested that compound **5d** can be used as a possible lead compound for the development of potential agrochemicals. Compounds **5d**, **5e**, **5f**, and **5i** showed ordinary activity against *S. sclerotiorum*, the EC_50_ values of 8.49 µg/mL, 14.97 µg/mL, 17.20 µg/mL, and 14.78 µg/mL, respectively, less effective than the positive control hymexazol (7.76 µg/mL). Compounds **5d**, **5e**, **5f**, and **5i** showed weak effectivity against *C. mandshurica*, with EC_50_ values of 79.92 µg/mL, 38.27 µg/mL, 98.18 µg/mL, and 44.50 µg/mL, respectively. Generally speaking, compounds **5d**, **5e**, **5f**, and **5i** showed weak to normal effective against *F. oxysporum*, *T. cucumeris*, *S. sclerotiorum*, *C. gloeosporioides*, *and P. infestans*, with EC_50_ values ranging from 14.73 µg/mL to 85.41 µg/mL. As indicated in Table 1 and Table 2, with *C. mandshurica* as the experimental target the relationship between antifungal activity and substituent attached to the benzene ring showed that a fluorinated phenyl group, such as compound **5e** (R = 2,6-2F), had better antifungal activity (97.8% fungicidal activity against *C. mandshurica* at 50 µg/mL, and the EC_50_ was 38.27 µg/mL).

**Figure 1:**
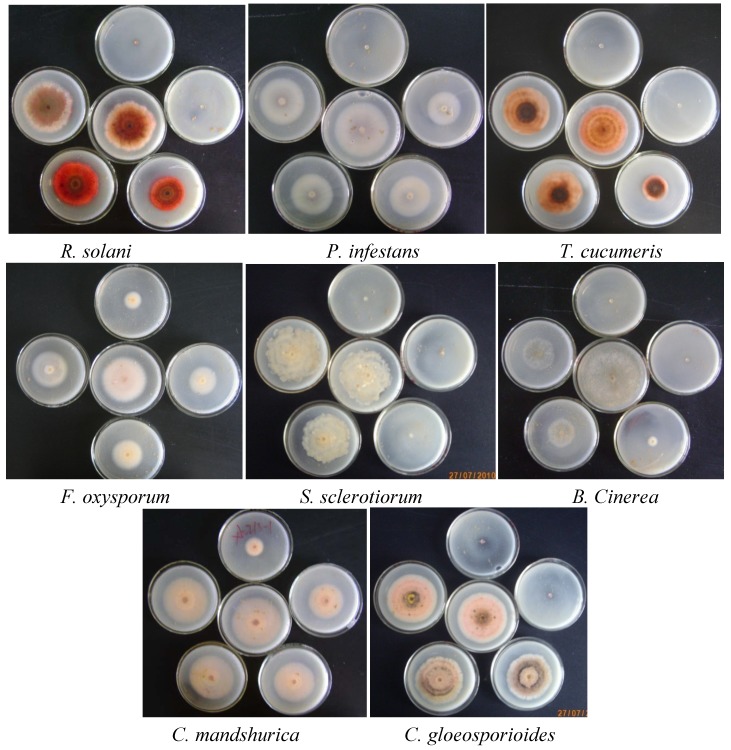
Effect of different concentrations of **5d** on the mycelial growth of pathogenic fungi (50, 25, 12.5, 6.25, 3.125, 0 µg/mL, the smaller of zone, the higher of concentration).

Compound **5d** had prominent antifungal activities against most of the tested fungi, and showed a broad-spectrum bioactivity; the inhibition effects of compound **5d** on mycelia growth *in vitro* at different concentrations are illustrated in Figure 1.

## 3. Experimental

### 3.1. General

Unless otherwise indicated, all common reagents and solvents were used as obtained from commercial suppliers without further purifications. The melting points of the products were determined on a XT-4 binocular microscope (Beijing Tech Instrument Co., China) and were not corrected. The IR spectra were recorded on a Bruker VECTOR 22 spectrometer in KBr disks. ^1^H- and ^13^C-NMR spectra (solvent CDCl_3_) were recorded on a JEOL-ECX 500 NMR spectrometer at room temperature using TMS as an internal standard, chemical shift values (*δ*) are given in parts per million. Elemental analysis was performed on an Elementar Vario-III CHN analyzer. Analytical TLC was performed on silica gel GF254. Column chromatographic purification was carried out using silica gel.

### 3.2. Preparation of the Intermediates **1–3**

Starting from the corresponding acid, and following the reported method [27,28], intermediates **3** were synthesized in three steps involving esterification, hydrazination, and cyclization. Specifically, 2-thiol-5-substituted-1,3,4-oxadiazole intermediates **3** were prepared by the reaction of substituted phenylhydrazide intermediates **2**, potassium hydroxide, and carbon disulfide in ethanol under reflux conditions. Intermediates **2** were synthesized from intermediates **1** and hydrazine hydrate in methanol under reflux condition. Intermediates **1** (substituted benzoic acid esters) were made through esterification reactions from the starting acids.

### 3.3. Preparation of the Intermediates **4**

To a solution of 2-thiol-5-substituted-1,3,4-oxadiazole **3** (2 mmol) and sodium hydroxide (0.08 g, 2 mmol) in water (20 mL), dimethyl sulfate (0.32 g, 2.5 mmol) was added dropwise. After stirring for 3 h at room temperature (20–23 °С), for solid products, the precipitate was filtered off, washed with 5% Na_2_CO_3_ solution and distilled water, dried and recrystallized from ethanol to afford the white solid products **4**; for liquid products, the reaction mixture was washed with 5% Na_2_CO_3_solution, and then extracted with ethyl ether (4 × 20 mL), the ethyl ether layer was dried with anhydrous Na_2_SO_4_, after removal of the organic solvent under reduced pressure, and oily products **4** were thus obtained.

*2-(Methylthio)-5-(2-(trifluoromethyl)phenyl)-1,3,4-oxadiazole *(**4a**): yield 79.2%; white solid; m.p. 76–78 °С; ^1^H-NMR *δ: *7.58–7.43 (m, 4H, benzene-H), 3.15 (s, 3H, SCH_3_); ^13^C-NMR *δ: *162.23, 159.43, 133.05, 132.78, 132.31, 131.67, 131.03, 122.34, 14.73.

*2-(Methylthio)-5-(2,3,4-trifluorophenyl)-1,3,4-oxadiazole *(**4b**): yield 77.9%;oil; ^1^H-NMR *δ: *7.88–7.46 (m, 2H, benzene-H), 3.22 (s, 3H, SCH_3_).

*2-(Methylthio)-5-(pyridin-4-yl)-1,3,4-oxadiazole *(**4c**): yield 75.4%; oil. ^1^H-NMR *δ: *8.66–7.47 (m, 4H, pyridin-H), 2.91 (s, 3H, SCH_3_).

*2-(Methylthio)-5-(2-fluorophenyl)-1,3,4-oxadiazole *(**4d**): yield 85.0%; oil; ^1^H-NMR *δ: *7.61–7.25 (m, 4H, benzene-H), 2.93 (s, 3H, SCH_3_); ^13^C-NMR *δ: *163.41, 159.94, 159.73, 136.37, 133.71, 132.32, 129.74, 121.45, 14.71.

*2-(Methylthio)-5-(2,6-difluorophenyl)-1,3,4-oxadiazole *(**4e**): yield 88.4%; oil; ^1^H-NMR *δ: *7.84–7.27 (m, 3H, benzene-H), 2.78 (s, 3H, SCH_3_); ^13^C-NMR *δ: *165.73, 164.15, 153.63, 151.74, 149.75, 123.50, 118.38, 116.23, 14.72.

*2-(Methylthio)-5-(2,6-dichlorophenyl)-1,3,4-oxadiazole *(**4f**): yield 78.3%; oil; ^1^H-NMR *δ: *7.63–7.24 (m, 3H, benzene-H), 2.81 (s, 3H, SCH_3_).

*2-(Methylthio)-5-(2-methoxyphenyl)-1,3,4-oxadiazole *(**4g**): yield 79.0%; oil; ^1^H-NMR *δ: *7.88–7.27 (m, 4H, benzene-H), 3.02 (s, 3H, OCH_3_), 2.79 (s, 3H, SCH_3_). ^13^C-NMR *δ: *165.23, 162.43, 138.69, 131.87, 128.77, 125.22, 122.37, 45.23, 14.43.

*2-(Methylthio)-5-(2-methylphenyl)-1,3,4-oxadiazole *(**4h**): yield 85.2%; oil; ^1^H-NMR *δ: *7.88–7.27 (m, 4H, benzene-H), 2.79 (s, 3H, SCH_3_), 2.68 (s, 3H, CH_3_); ^13^C-NMR *δ: *166.14, 161.67, 138.34, 131.92, 128.80, 126.21, 121.43, 22.13, 14.68.

*2-(Methylthio)-5-(2-bromophenyl)-1,3,4-oxadiazole *(**4i**): yield 85.3%; oil; ^1^H-NMR *δ: *7.81–7.44 (m, 4H, benzene-H), 3.03 (s, 3H, SCH_3_).

*2-(Methylthio)-5-(2-chloro-6-fluorophenyl)-1,3,4-oxadiazole *(**4j**): yield 79.3%; oil; ^1^H-NMR *δ: *7.94–7.33 (m, 3H, benzene-H), 2.71 (s, 3H, SCH_3_).

### 3.4. Preparation of the Title Compounds **5**

A solution of 2-methylthio-5-substituted-1,3,4-oxadiazole **4** (3.83 mmol) in glacial acetic acid (10 mL) was treated dropwise at 10 °C with potassium permanganate (0.78 g, 4.98 mmol) as a 5% aqueous solution over 20 min. The reaction was allowed to proceed for an additional 20 min, 40% strength aqueous sodium hydrogen sulphite solution was subsequently added until the mixture was decolorized, and diluted with 50 mL of water. The product was filtered and recrystallised from ethanol give the title compounds **5**.

*2-(Methylsulfonyl)-5-(2-(trifluoromethyl)phenyl)-1,3,4-oxadiazole *(**5a**): yield 86.6%; white solid; m.p. 102–104 °С; ^1^H-NMR *δ: *7.66–7.14 (m, 4H, ArH), 3.54 (s, 3H, CH_3_); ^13^C-NMR *δ: *162.81, 161.81, 135.43, 135.25, 133.65, 133.29, 112.86, 110.50, 43.12; IR (cm^−1^) *ν: *2931, 1616, 1558, 1543, 1458, 1338, 1192; Anal. Calcd for C_10_H_7_F_3_N_2_O_3_S: C 41.10, H 2.41, N 9.59; found: C 41.46, H 2.02, N 9.88.

*2-(Methylsulfonyl)-5-(2,3,4-trifluorophenyl)-1,3,4-oxadiazole *(**5b**): yield 84.3%; white solid; m.p. 114–115 °С; ^1^H-NMR *δ: *7.97–7.32 (m, 2H, ArH), 3.54 (s, 3H, CH_3_); ^13^C-NMR *δ: *167.56, 161.52, 161.47, 125.65, 123.74, 121.74, 119.78, 111.23, 43.11; IR (cm^−1^) *ν: *3027, 2926, 1616, 1558, 1506, 1350, 1156; Anal. Calcd for C_9_H_5_F_3_N_2_O_3_S: C 38.85, H 1.81, N 10.07; found: C 38.56, H 1.51, N 9.82.

*2-(Methylsulfonyl)-5-(pyridin-4-yl)-1,3,4-oxadiazole *(**5c**): yield 84.0%; white solid; m.p. 134–136 °С; ^1^H-NMR *δ: *8.41–7.27 (m, 4H, pyridine-H), 3.54 (s, 3H, CH_3_); ^13^C-NMR *δ: *167.38, 162.45, 151.12, 150.45, 133.12, 121.15, 112.40, 40.32; IR (cm^−1^) *ν: *3031, 2927, 1616, 1558, 1373, 1153; Anal. Calcd for C_8_H_7_N_3_O_3_S: C 42.66, H 3.13, N 18.66; found: C 41.29, H 2.97, N 19.01.

*2-(Methylsulfonyl)-5-(2-fluorophenyl)-1,3,4-oxadiazole *(**5d**): yield 83.7%; white solid; m.p. 97–99 °С; ^1^H-NMR *δ: *8.12–7.29 (m, 4H, ArH), 3.54 (s, 3H, CH_3_); ^13^C-NMR *δ: *162.30, 161.58, 159.52, 135.34, 135.27, 130.35, 125.07, 117.50, 117.33, 110.85, 43.08; IR (cm^−1^) *ν: *3012, 2927, 1616, 1541, 1458, 1340, 1145; Anal. Calcd for C_9_H_7_FN_2_O_3_S: C 44.63, H 2.91, N 11.56; found: C 44.41, H 2.68, N 11.72.

*2-(Methylsulfonyl)-5-(2,6-difluorophenyl)-1,3,4-oxadiazole *(**5e**): yield 80.2%; white solid; m.p. 131–132 °С; ^1^H-NMR *δ: *7.63–7.12 (m, 3H, ArH); 3.53 (s, 3H, CH_3_); ^13^C-NMR *δ: *162.86, 162.02, 159,94, 159.17, 135.25, 112.89, 112.73, 43.12; IR (cm^−1^) *ν: *3022, 2931, 1629, 1587, 1477, 1352, 1153; Anal. Calcd for C_9_H_6_F_2_N_2_O_3_S: C 41.54, H 2.32, N 10.77; found: C 41.41, H 2.01, N 10.48.

*2-(Methylsulfonyl)-5-(2,6-dichlorophenyl)-1,3,4-oxadiazole *(**5f**): yield 84.3%; white solid; m.p. 126–128 °С; ^1^H-NMR *δ: *7.51 (s, 3H, ArH), 3.55 (s, 3H, CH_3_); ^13^C-NMR *δ: *163.37, 162.00, 136.65, 134.00, 128.62, 128.44, 122.53, 43.13; IR (cm^−1^) *ν: *3028, 2927, 1616, 1587, 1473, 1372, 1153; Anal. Calcd for C_9_H_6_Cl_2_N_2_O_3_S: C 36.88, H 2.06, N 9.56; found: C 36.82, H 1.97, N 9.71.

*2-(Methylsulfonyl)-5-(2-methoxyphenyl)-1,3,4-oxadiazole *(**5g**): yield 87.2%; white solid; m.p. 127–129 °С; ^1^H-NMR *δ: *8.02–7.36 (m, 4H, ArH), 3.54 (s, 3H, CH_3_), 2.74 (s, 3H, OCH_3_); ^13^C-NMR *δ: *155.79, 155.32, 132.81, 131.83, 131.36, 129.76, 128.26, 122.49, 43.01, 22.21; IR (m^−1^) *ν: *3084, 2827, 1635, 1558, 1506, 1458, 1379, 1163; Anal. Calcd for C_10_H_10_N_2_O_4_S: C 47.24, H 3.96, N 11.02; found: C 47.19, H 4.28, N 10.96.

*2-(Methylsulfonyl)-5-(2-methylphenyl)-1,3,4-oxadiazole *(**5h**): yield 83.4%; white solid; m.p. 117–119 °С; ^1^H-NMR *δ: *8.00–7.08 (m, 4H, ArH), 3.99 (s, 3H, CH_3_), 3.52 (s, 3H, CH_3_); ^13^C-NMR *δ: *165.66, 161.94, 158.56, 134.76, 131.14, 121.01, 112.16, 56.17, 43.04; IR (KBr, cm^−1^) *ν: *3010, 2926, 1604, 1533, 1498, 1338, 1155; Anal. Calcd for C_10_H_10_N_2_O_3_S: C 50.41, H 4.23, N 11.76; found: C 50.03, H 4.45, N 11.49.

*2-(Methylsulfonyl)-5-(2-bromophenyl)-1,3,4-oxadiazole *(**5i**): yield 78.8%; white solid; m.p. 134–135 °С; ^1^H-NMR *δ: *8.00–7.46 (m, 4H, ArH), 3.54 (s, 3H, CH_3_);^13^C-NMR *δ: *165.54, 162.55, 135.02, 133.87, 132.22, 127.96, 123.57, 122.29, 43.07; IR (cm^−1^) *ν: *3028, 2943, 1615 1557, 1516, 1443, 1373, 1155; Anal. Calcd for C_9_H_7_BrN_2_O_3_S: C 35.66, H 2.33, N 9.24; found: C 35.87, H 2.44, N 9.61.

*2-(Methylsulfonyl)-5-(2-chloro-6-fluorophenyl)-1,3,4-oxadiazole *(**5j**): yield 86.1%; white solid; m.p. 121–123 °С; ^1^H-NMR *δ: *8.89–7.45 (m, 3H, ArH), 3.543 (s, 3H, CH_3_); ^13^C-NMR *δ: *165.91, 161.87, 134.71, 134.65, 132.54, 131.23, 127.38, 42.95; IR (cm^−1^) *ν: *3033, 2941, 1615, 1557, 1456, 1337, 1151; Anal. Calcd for C_9_H_6_ClFN_2_O_3_S: C 39.07, H 2.19, N 10.13; found: C 39.02, H 2.29, N 10.11.

### 3.5. Antifungal Activities Test

The antifungal activities were tested against two pathogenic fungi, *Fusarium oxysporum* and *Cytospora mandshurica,* by the poison plate technique [29]. Compounds were dissolved in dimethyl sulfoxide (1 mL) before mixing with potato dextrose agar (PDA, 90 mL). The compounds were tested at a concentration of 50 μg/mL. All fungi were incubated in PDA at 27 ± 1 °C for 4 days to get new mycelium for antifungal assay. Then mycelia dishes of approximately 4 mm diameter were cut from culture medium, and one of them was picked up with a sterilized inoculation needle and inoculated in the center of PDA plate aseptically. The inoculated plates were incubated at 27 ± 1 °C for 5 days. DMSO in sterile distilled water served as negative control, while hymexazol acted as positive control. For each treatment, three replicates were conducted. The radial growth of the fungal colonies was measured and the data were statistically analyzed. The inhibitory effects of the test compounds *in vitro *on these fungi were calculated by the formula: *I*(%) = [(*C* − *T*)/(*C* − 0.4)] × 100, where *C* represents the diameter of fungi growth on untreated PDA, and *T* represents the diameter of fungi on treated PDA while *I* means the inhibition rate. 

Some of the title compounds were tested against eight pathogenic fungi namely *F. oxysporum*, *C. mandshurica*, *Phytophthora infestans*, *Rhizoctonia solani*, *Thanatephorus cucumeris*, *Colletotrichum gloeosporioides*, *Botrytis cinerea* and *Sclerotinia sclerotiorum* at different concentrations of 50, 25, 12.5, 6.25, 3.125, 0 µg/mL. The EC_50_ (effective dose for 50% inhibition µg/mL) values were estimated statistically by probit analysis with the help of the probit package of the SPSS software using a personal computer. The average EC_50_ was taken from at least three separate analyses for inhibition of growth using the basic EC_50_ program version SPSS 11.5.

## 4. Conclusions

In summary, a series of new sulfone compounds containing 1,3,4-oxadiazole moieties and based on the lead compound **Ia** was designed and synthesized. The title compounds showed promising antifungal activities against some typical fungi often occurring in the Chinese agro-ecosystem, including *F. oxysporum*, *C. mandshurica*, *R. solani*, *T. cucumeris*, *S. sclerotiorum*, *C. gloeosporioides*, *P. infestans*, *B. cinerea.* Among them, some compounds showed superiority over the lead compound **Ia**, such as compound **5b** with EC_50_ values ranging from 5.21 μg/mL to 79.2 μg/mL, and some, such as compounds **5b**, **5d** and **5e** showed superiority over the commercial fungicide hymexazol. In particular, compounds **5d**, **5e**, **5f**, and **5i** showed prominent activity against *B. cinerea*, with EC_50_ values of 5.21 µg/mL, 8.25 µg/mL, 8.03 µg/mL, and 21.00 µg/mL, respectively. These results demonstrated that some compounds such as **5d** can be used as possible lead compounds for the development of potential agrochemicals.

## References

[1] Russell P.E. (2005). A century of fungicide evolution. J. Agric. Sci..

[2] Copping L.G., Menn J.J. (2000). Biopesticides: A review of their action, applications and efficacy. Pest. Manag. Sci..

[3] Fitzjohn S., Robinson M.P. (1994). Benzoxazole and benzothiazole derivatives. WO 9406783, 1994. Chem. Abst..

[4] Hiromichi I., Masakazu T., Ten U., Seiichi K. (1994). Preparation of disulfonylthiadiazoles and their use as agrochemical microbicides. JP 94116252, 1994. Chem. Abst..

[5] Plant A., Boehmer J.E., Black J., Sparks T.D. (2006). Isoxazoline derivatives and their preparation, herbicidal composition, and use as herbicides to control weeds or plant growth inhibition. WO 2006024820, 2006. Chem. Abst..

[6] Gong P., Chai H.F., Zhao Y.F., Zhao C.S. (2006). Synthesis and *in vitro* anti-hepatitis B virus activities of some ethyl 5-hydroxy-1*H*-indole-3-carboxylates. Bioorg. Med. Chem..

[7] Tai X.S., Yin X.H., Tan M.Y. (2003). Crystal structure and antitumor activity of tri[2-[*N*-(4'-methyl-benzylsulfonyl)amino]ethyl]-amine. Chin. J. Struct. Chem..

[8] Fang S.H., Padmavathi V., Rao Y.K., Subbaiah D.R.C., Thriveni P., Geethangili M., Padaja A., Tzeng Y.M. (2006). Biological evaluation of sulfone derivatives as anti-inflammatory and tumor cells growth inhibitory agents. Int. Immunopharmacol..

[9] Vedula M.S., Pulipaka A.B., Venna C., Chintakunta V.K., Jinnapally S., Kattuboina V.A., Vallakati R.K., Basetti V., Akella V., Rajgopai S. (2003). New styryl sulfones as anticancer agents. Eur.J.Med.Chem..

[10] Silvestri R., Artico M., Regina G.L. (2004). Anti-HIV-1 activity of pyrryl aryl sulfone (PAS) derivatives: Synthesis and SAR studies of novel esters and amides at the position 2 of the pyrrole nucleus. Farmaco.

[11] Talath S., Gadad A.K. (2006). Synthesis, antibacterial and anti-tubercular activities of some 7-[4-(5-amino-[1,3,4]thiadiazole-2-sulfonyl)-piperazin-1-yl] fluoroquinolonic derivatives. Eur. J. Med. Chem..

[12] Diehr H.J., Marhold A., Brandes W., Hanssler G. (1990). Preparation of 2,5-bis (alkylsulfonyl)-1,3,4-thiadiazoles as agrochemical fungicides. DE 3838432, 1990. Chem. Abst..

[13] Assmann L., Stenzel K., Erdelen C., Kugler M., Wachtler P. (2002). Nitrophenyl sulfonyl imidazoles and use thereof for controlling vegetable and animal pests. US 20020094936 A1, 2002. Chem. Abst..

[14] Yuan D.K., Li Z.M., Zhao W.G., Chen H.S. (2003). Synthesis and bioactivity of 2-substituted amino-5-pyrazolyl-1,3,4-oxadiazoles. Chin. J. Appl. Chem..

[15] Ohshima T., Komyojia T., Mitani S. (2004). Development of a novel fungicide, eyazofamid. J. Pestic. Sci..

[16] Komyji N., Terumasa K., Kazumi S., Keiichiro I. (1989). Imidazole compounds and biocidal compositions comprising the same. EP 0298196A1, 1989. Chem. Abst..

[17] Liu Z., Yang G., Qin X. (2001). Syntheses and biological activities of novel diheterocyclic compounds containing 1,2,4-triazolo[1,5-α]pyrimidine and 1,3,4-oxadiazole. J. Chem. Technol. Biotechnol..

[18] Jiang L., Tan Y., Zhu X., Wang Z., Zuo Y., Chen Q., Xi Z., Yang G.F. (2010). Design, synthesis, and 3D-QSAR analysis of novel 1,3,4-oxadiazol-2(3*H*)-ones as protoporphyrinogen oxidase inhibitors. J. Agric. Food Chem..

[19] Qian X., Zhang R. (1996). Syntheses and insecticidal activities of novel 2,5-disubstituted-1,3,4-oxadiazoles. J. Chem. Tech. Biotechnol..

[20] Cao S., Qian X., Song G., Chai B., Jiang Z. (2003). Synthesis and antifeedant activity of new oxadiazolyl 3(2*H*)-pyridazinones. J. Agric. Food Chem..

[21] Keshari K.J., Abdul S., Yatendra K., Mohd S., Ratan L.K., Jainendra J., Vikash K., Priyanka S. (2010). Design, synthesis and biological evaluation of 1,3,4-oxadiazole derivatives. Eur. J. Med. Chem..

[22] Chen C.J., Song B.A., Yang S., Xu G.F., Bhadury P.S., Jin L.H., Hu D.Y., Li Q.Z., Liu F., Xue W. (2007). Synthesis and antifungal activities of 5-(3,4,5-trimethoxyphenyl)-2-sulfonyl-1,3,4-thiadiazole and 5-(3,4,5-trimethoxyphenyl)-2-sulfonyl-1,3,4-oxadiazole derivatives. Bioorg. Med. Chem..

[23] Liu F., Luo X.Q., Song B.A., Bhadury P.S., Yang S., Jin L.H., Xue W., Hu D.Y. (2008). Synthesis and antifungal activity of novel sulfoxide derivatives containing trimethoxyphenyl substituted 1,3,4-thiadiazole and 1,3,4-oxadiazole moiety. Bioorg. Med. Chem..

[24] Pees B., Paul J.M., Oget N., Sindt M., Mieloszynski J.L. (2003). Synthesis of fluoro-substituted monomers bearing a functionalised lateral chain: Part 2. Preparation of sulfoxides and sulfones containing monomers. J. Fluorine Chem..

[25] Yamazaki S. (1996). Selective synthesis of sulfones and sulfoxides by methytrioxorhenium catalyzed oxidation of sulfides with hydrogen peroxide. Bull. Chem. Soc. Jpn..

[26] Confalone P.N., Woodward R.B. (1983). A novel synthesis of peptides based on the photochemistry of 5-azido-1,3,4-oxadiazoles. J. Am. Chem. Soc..

[27] Song B.A., Chen C.J., Yang S., Jin L.H., Xue W., Zhang S.M., Zou Z.H., Hu D.Y. (2005). Synthesis, structure and antitumor activity of 2-alkylthio-5-(3,4,5-trimethoxyphenyl)-1,3,4-thiadiazole compounds. Acta Chim. Sinica.

[28] Chen C.J., Song B.A., Yang S., Xu G.F., Bhadury P.S., Jin L.H., Hu D.Y., Li Q.Z., Liu F., Xue W. (2007). Synthesis and antifungal activities of 5-(3,4,5-trimethoxyphenyl)- 2-sulfonyl-1,3,4-thiadiazole and 5-(3,4,5-trimethoxyphenyl)-2-sulfonyl-1,3,4-oxadiazole derivatives. Bioorg. Med. Chem..

[29] Tarun K.C., Prem D.J. (2006). Antifungal activity of 4-methyl-6-alkyl-2*H*-pyran-2-ones. J. Agric. Food Chem..

